# Protective potential of royal jelly against hydroxyurea -induced hepatic injury in rats via antioxidant, anti-inflammatory, and anti-apoptosis properties

**DOI:** 10.1371/journal.pone.0265261

**Published:** 2022-03-18

**Authors:** Hossam G. Tohamy, Mahmoud S. El-Neweshy, Mohamed Mohamed Soliman, Samy Sayed, Mustafa Shukry, Heba I. Ghamry, Hoda Abd-Ellatieff

**Affiliations:** 1 Department of Pathology, Faculty of Veterinary Medicine, Alexandria University, Alexandria, Egypt; 2 Department of Pathology, Faculty of Veterinary Medicine, Kafrelsheikh University, Kafrelsheikh, Egypt; 3 Department of Clinical Laboratory Sciences, Turabah University College, Taif University, Taif, Saudi Arabia; 4 Department of Science and Technology, University College-Ranyah, Taif University, Taif, Saudi Arabia; 5 Department of Physiology, Faculty of Veterinary Medicine, Kafrelsheikh University, Kafrelsheikh, Egypt; 6 Home Economics College, King Khalid University, Abha, Saudi Arabia; 7 Research Center for Advanced Materials Science (RCAMS), King Khalid University, Abha, Saudi Arabia; 8 Department of Pathology, Faculty of Veterinary Medicine, Damanhour University, Damanhour, Egypt; Zagazig University, EGYPT

## Abstract

Hydroxyurea (HDU) is a widely used medication for various malignancies, thalassemia, and sickle cell anemia with reported side effects. The current study investigated HDU- induced hepatic injury and the protective potential of the royal jelly (RJ) against this hepatotoxic effect in the light of hepatic oxidative/ antioxidative status, pro-inflammatory cytokine, apoptosis signaling pathway, and histopathology. Sixty albino rats were used (n = 10/group) for 60 days: control, RJ (100 mg/kg body weight, orally), HDU (225 mg/kg body weight, orally), 2HDU (450 mg/kg body weight, orally), and HDU + RJ groups. HDU-treated rats showed significant elevation of liver function tests as aspartate aminotransferase, alanine aminotransferase, and alkaline phosphatase, as well as malondialdehyde and nitric oxide (oxidative biomarkers) and significant decreased hepatic antioxidant molecules (reduced glutathione, superoxide dismutase, and glutathione peroxidase), compared to a control group, that more pronounced in the high dose of HDU. In addition, HDU induced significant upregulation of TNF-α and the Caspase-3 apoptotic pathway. Moreover, the liver of HDU treated groups showed various hepatic lesions from mild to severe necrotic changes related to the HDU dose. However, administration of RJ with HDU improved liver function tests, liver histology, and hepatic oxidative/antioxidative status concerning HDU groups. Furthermore, oral RJ administration with HDU significantly lessens the immune-expression area % of TNF-α and Caspase-3. Thus, the royal jelly has antioxidant, anti-inflammatory, and anti-apoptotic properties against HDU- induced hepatic injury and could be, therefore, used as adjuvant therapy in patients with long-term HDU medication.

## 1. Introduction

Hydroxyurea (HU), a united states food and drug administration-approved drug [1], is an inhibitor of ribonucleotide reductase frequently used to treat myeloproliferative illnesses and sickle cell anemia [2]. In addition, HUD is used as an anti-tumor drug to treat various malignancies [3] such as Melanoma; Leukemia; Ovarian cancer; Head and neck cancer [4]. It is being utilized as an antineoplastic agent in the treatment of malignant melanoma, head and neck malignancies, brain tumors, and a few non-malignant illnesses in combination with radiation therapy [5, 6].

Furthermore, it is recurrently used to treat HIV infections and sickle cell disease, polycythemia vera, and essential thrombocytopenia [1, 7, 8]. Although its advantageous effect, there are many reports on gonadotoxicity [3, 9] cytotoxicity [10], and genotoxicity impact of HDU [11, 12]. The integrated mechanism of HDU-induced gonadotoxicity (decreased sperm production and spermatogenic arrest, and reduced oocyte maturation) and cytotoxicity is explained by the overproduction of the formation of the reactive oxygen species [3, 9]. Also, HDU induced apoptosis in fetal tissues [13, 14] and cancer cell lines [15, 16]. Furthermore, HDU triggered microsomal activation-dependent mutagenicity [17, 18]. In addition, several reports describe the hepatotoxic effect of HDU, including hepatitis [19], hepatic dysfunction [20], and acute elevation of liver function tests [21]; however, the mechanism of this effect has not been thoroughly studied previously.

Royal jelly (RJ) is a thick milky-white material secreted by young, newly emerged honeybee workers [22]. Its ingredients consist of 60–70% water, 9–18% protein, and 10–16% total sugars with a mixture of small amounts of free amino acids, vitamins, salts, and lipids [23]. Several valuable properties of RJ have been reported, including anti-inflammatory, antineoplastic, hypotensive effects, and accelerated immune response [24, 25]. Furthermore, several studies have evidenced that RJ has antioxidant [26, 27] and hepatoprotective effects [28, 29].

The current study aims to investigate the hepatotoxic effect of HDU in light of hepatic oxidative/ antioxidative status, a pro-inflammatory cytokine, apoptotic signaling pathway, and histopathology. In addition, the protective potential of RJ against HDU induced liver injury was also studied.

## 2. Materials and methods

### 2.1 Chemicals and reagents

Hydroxyurea (Hydrea^®^) capsules containing 500 mg hydroxycarbamide as the active ingredient (E.R. Squibb & Sons Ltd., England). Immediately before administration, HDU and RJ were dissolved in distilled water. Royal jelly (Royal Jelly^®^ capsules containing 340 mg lyophilized royal jelly equivalent to 1000-mg-crude royal jelly) was also obtained from Pharco Pharmaceuticals Company (Cairo, Egypt).

### 2.2 Experimental animals and animal welfare

Sixty male Wistar rats (190 ± 10 g), 3–4 months old, were obtained from Medical Research Institute (Alexandria, Egypt). The experiment was permitted by the Research Ethics Committee of Alexandria University, following the National Institutes of Health guidelines for the care and use of laboratory animals [30]. All rats were raised in plastic cages and fed a standard balanced diet and water ad libitum throughout the experiment.

### 2.3 Experimental design

Following an acclimatization period of 14 days, the experimental animals were randomly allocated into six groups (n = 10/ each). All rats were treated orally using the stomach tube daily for 60 successive days: 1) Control group was administered 2 ml saline solution. 2) RJ group was given 100-mg royal jelly/ kg body weight (bwt) [31, 32]. 3) HDU group received a therapeutic dose of 225 mg of HDU / kg body weight [3, 33]. 4) 2HDU group received a double therapeutic dose of 450 mg of HDU / kg body weight [3]. 5) RJ + HDU group received 100 mg/kg bwt royal jelly plus 225 mg/kg body weight HDU. 6) RJ + 2HDU group received 100 mg/kg bwt royal jelly plus 450 mg/kg body weight HDU.

### 2.4 Samples collection and processing

Twenty-four hours following the last treatment, rats were anesthetized with sodium pentobarbital (30 mg kg^−1^, i.p.) [34, 35]. Samples were collected from the retro-orbital venous plexus. Serum was separated and kept under at − 4°C for the subsequent biochemical analysis of liver enzymes. Following euthanization by intraperitoneal injections of 80 mg/kg of ketamine (Nimatek; Eurovet, Bladel, the Netherlands) combined with 0.5mg/kg of medetomidine (Domitor, Novartis, Arnhem, The Netherlands) [36]. The abdomen was opened, part of the liver of all rats was collected and fixed for at least 48 hours in 10% buffered formalin for the subsequent immunohistochemical and histopathological examinations. Another part of the liver of all rats was preserved at − 20°C for assay of oxidative and anti-oxidative parameters.

### 2.5 Biochemical analysis of serum hepatocellular enzymes

The level of serum hepatocellular enzymes, including aspartate aminotransferase (AST), alanine aminotransferase (ALT), and alkaline phosphatase (ALP), were measured using the kits of Diamond Diagnostics (Egypt) according to manufacturers’ directions. AST and ALT were measured as described previously [37], while ALP level was measured previously [38].

### 2.6 Oxidative stress and antioxidant capacity assay

The liver homogenate was prepared as described previously [3, 39]. In the liver homogenate, the biomarkers of oxidative stress were measured, including lipid peroxidation biomarker as malondialdehyde (MDA) and nitric oxide (NO) in the aliquots of the hepatic supernatant as described by [40] and [41], respectively. In addition, reduced glutathione (GSH), glutathione peroxidase (GPx), and superoxide dismutase (SOD) levels were measured as described previously [42–44], respectively.

### 2.7 Caspase-3 and TNF-α immunostaining

Deparaffinized liver sections were used for immunostaining of TNF-α and Caspase-3 as described previously [45]. Caspase-3 immunostaining was done using rabbit polyclonal anti-cleaved Caspase-3 antibody diluted 1:100 (BioCare Medical, Cat. CP229C, Concord, CA, USA). TNF-α immunostaining was done using a polyclonal rabbit anti-TNFα antibody diluted 1: 400 (GeneTex, Cat# GTX110520). Twenty sections were examined in each group. To determine the Caspase-3 and TNF-α immunoreactivity, the DAB-stained cytoplasmic option in the IHC profiler Image-J plugin was used as described previously [46] in the captured photomicrographs. Finally, the area% of Caspase-3 and TNF-α expressions was measured using Image J software (freely available public domain image processing software) as described previously [47].

### 2.8 Hepatic histopathology

Using the paraffin-embedding technique, the fixed liver samples of all rats were processed. The obtained 4–5 μm thick sections were stained with hematoxylin and eosin [48]. Finally, the stained liver slides were examined under a light microscope to evaluate the histopathological changes. The histopathological scoring was performed by measuring the area% of the affected tissues using Image J software as described previously [47].

### 2.9 Statistical analysis

Statistical analysis was done by GraphPad Prism version 7.0 for Windows (GraphPad Software Inc., San Diego, USA). The obtained data were performed by one-way analysis of variance (ANOVA) followed with Dunnett’s multiple comparison test to determine the significance of each treated group versus the control group (as negative control). In addition, the outcome of oral royal jelly administration in RJ + HDU and RJ + 2HDU groups was compared to HDU and 2HDU groups, respectively (as positive control) using an unpaired t-test (two-tailed). The analyzed data are presented as the mean ± standard error (SEM). Mean values were considered statistically significant when p < 0.05.

## 3. Results

### 3.1 Survival rate and body weight changes

**Fig 1:**
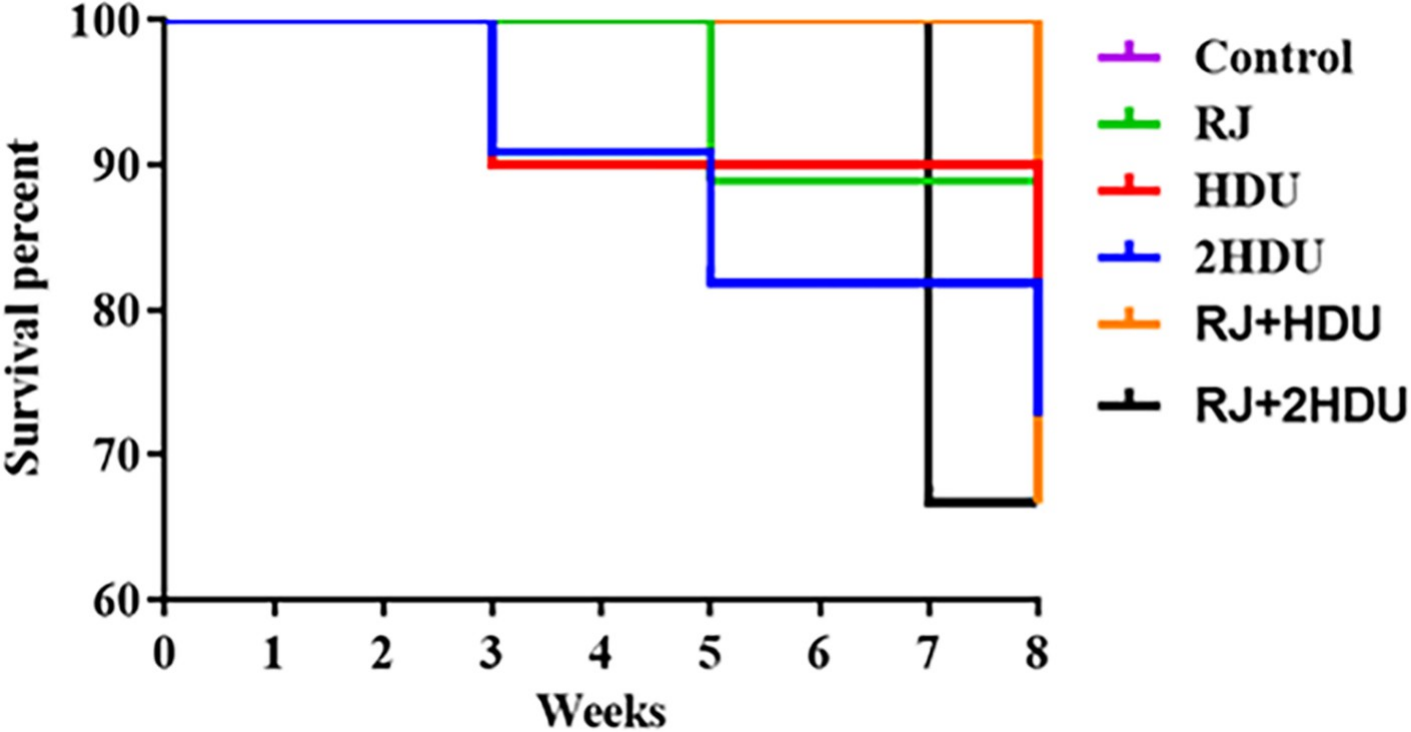
The survival rate.

**Fig 2:**
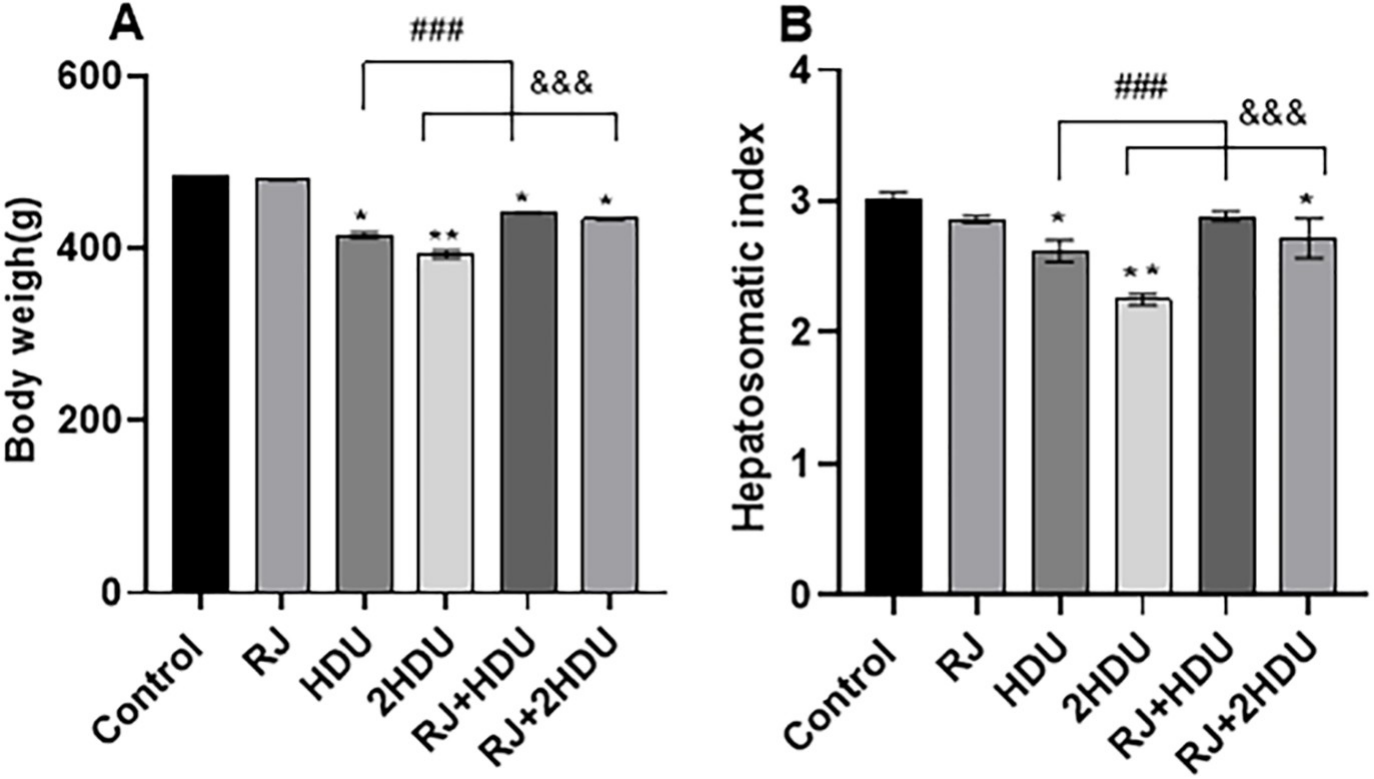
Effects of oral royal jelly (RJ; 100 mg/kg bwt) administration on body weight (A) and hepatosomatic index (B) in rats treated with hydroxyurea (HDU; 225 or 450 mg/kg bwt) for 60 days. Data are presented as the mean ± SEM. Compared to the control, significance at (p < 0.001) *** (ANOVA with Dunnett’s multiple comparison test). Compared to HDU treated groups using Unpaired t-test, significance at p < 0.01 ## and p < 0.001 &&& for HDU and 2HDU groups, respectively).

Each week during the experimental period, the number of animals that died was recorded, as was the survival rate of the control group (88.8%); In the fifth week, one rat died as a result of medication perfusion into the esophagus via stomach tube. Survival % for the groups were as follows: RJ: 88.8%; HDU: 80. %; 2HDU: 72.72%; RJ+HDU: 66.6%; RJ+2HDU: 66.6% (Fig 1). As shown in Fig 2(A) and 2(B), a significant decrease (p < 0.001) in bodyweight and hepatosomatic index in rats treated with HDU in HDU and 2HDU groups compared to the control group. In addition, the body weight and the hepatosomatic index were significantly increased in the RJ + HDU and RJ+2HDU groups to the HDU and 2HDU groups, respectively.

### 3.2 Serum hepatocellular enzymes

**Fig 3:**
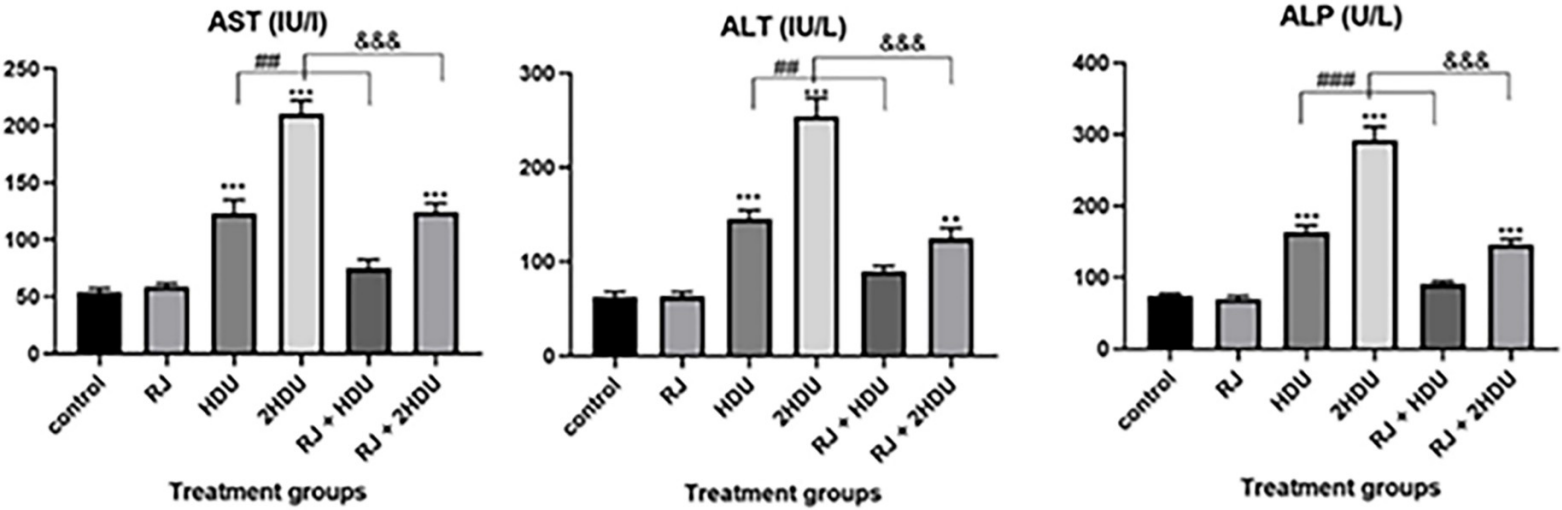
Effects of oral royal jelly (RJ; 100 mg/kg bwt) administration on serum aspartate aminotransferase (AST), alanine aminotransferase (ALT), and alkaline phosphatase (ALP) levels in rats treated with hydroxyurea (HDU; 225 or 450 mg/kg bwt) for 60 days. Data are presented as the mean ± SEM. Compared to the control, significance at (p < 0.01) ** and (p < 0.001) *** (ANOVA with Dunnett’s multiple comparison test). Compared to HDU treated groups using Unpaired t-test, significance at p < 0.001 ### and &&& for HDU and 2HDU groups, respectively).

As shown in Fig 3, a significant increase (p < 0.001) in the level of serum hepatocellular enzymes (AST, ALT, and ALP) in rats treated with HDU in HDU and 2HDU groups compared to the control group. In addition, rats treated with a therapeutic dose of HDU showed nearly double the fold of serum hepatocellular enzymes compared to the control group. Meanwhile, a double dose of HDU showed increased serum hepatocellular enzymes by about four-fold. Interestingly, the level of serum hepatocellular enzymes was significantly decreased (p < 0.001) in the RJ + HDU group to the control group level and by about 40% in the RJ + 2HDU group.

### 3.3 Hepatic oxidative stress

**Fig 4:**
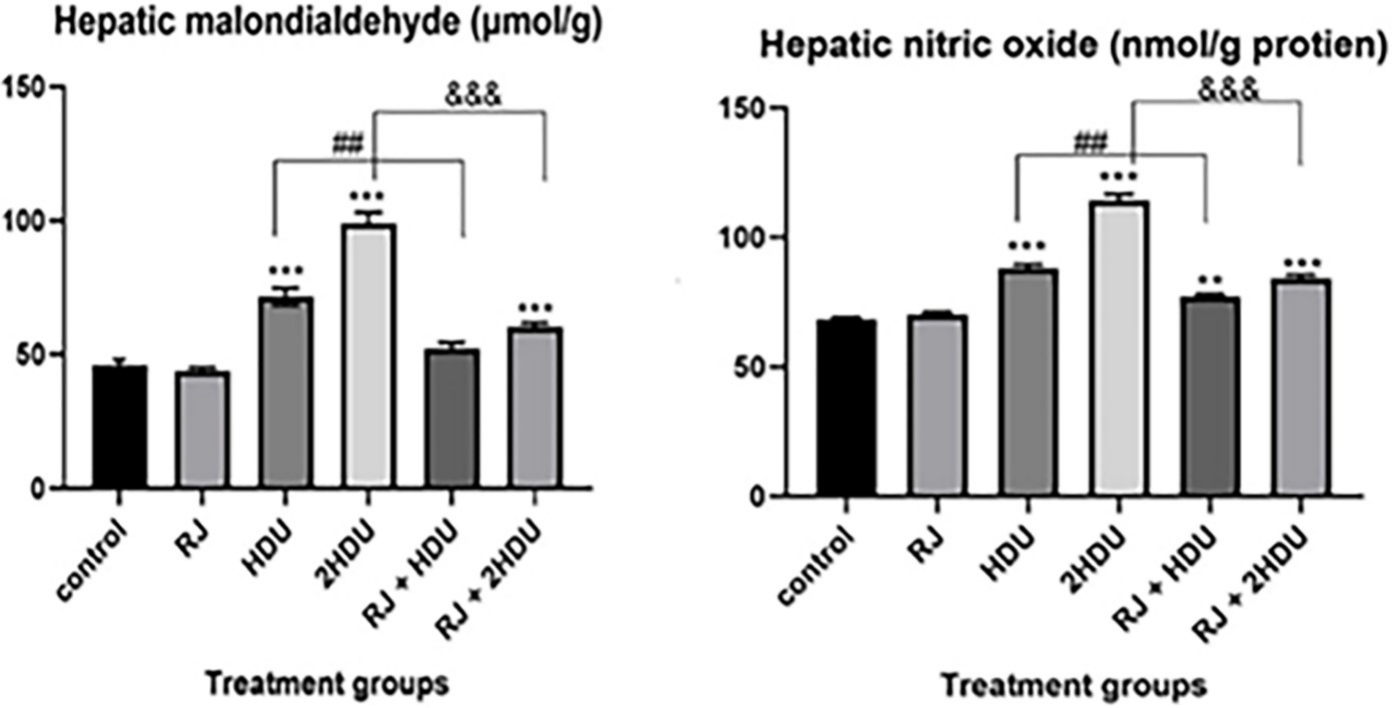
Effects of oral royal jelly (RJ; 100 mg/kg bwt) administration on the biomarkers of oxidative damage, malondialdehyde (MDA), and nitric oxide (NO) formation in rats treated with hydroxyurea (HDU; 225 or 450 mg/kg bwt) for 60 days. Data are presented as the mean ± SEM. Compared to the control, significance at (p < 0.01) ** and (p < 0.001) *** (ANOVA with Dunnett’s multiple comparison test). Compared to HDU treated groups using Unpaired t-test, significance at p < 0.01 ## and p < 0.001 &&& for HDU and 2HDU groups, respectively).

The results of hepatic oxidative stress of HDU and the effect of RJ in control and treated rats were presented in Fig 4 HDU induced statically (p < 0.001) elevation in hepatic MDA and NO, which is more pronounced in the high dose HDU compared to the control group. Rats received RJ + HDU induced a significant (p < 0. 001) decrease of MDA content to the control group level but showed a significant decrease (p < 0. 001) in NO by about 26%. Furthermore, RJ treatment in rats that received a double dose of HDU significantly alleviated (p < 0.001) the harmless effect of HDU on hepatic MDA and No by 39.3% and 26.3%, respectively.

### 3.4 Hepatic antioxidative status

**Fig 5:**
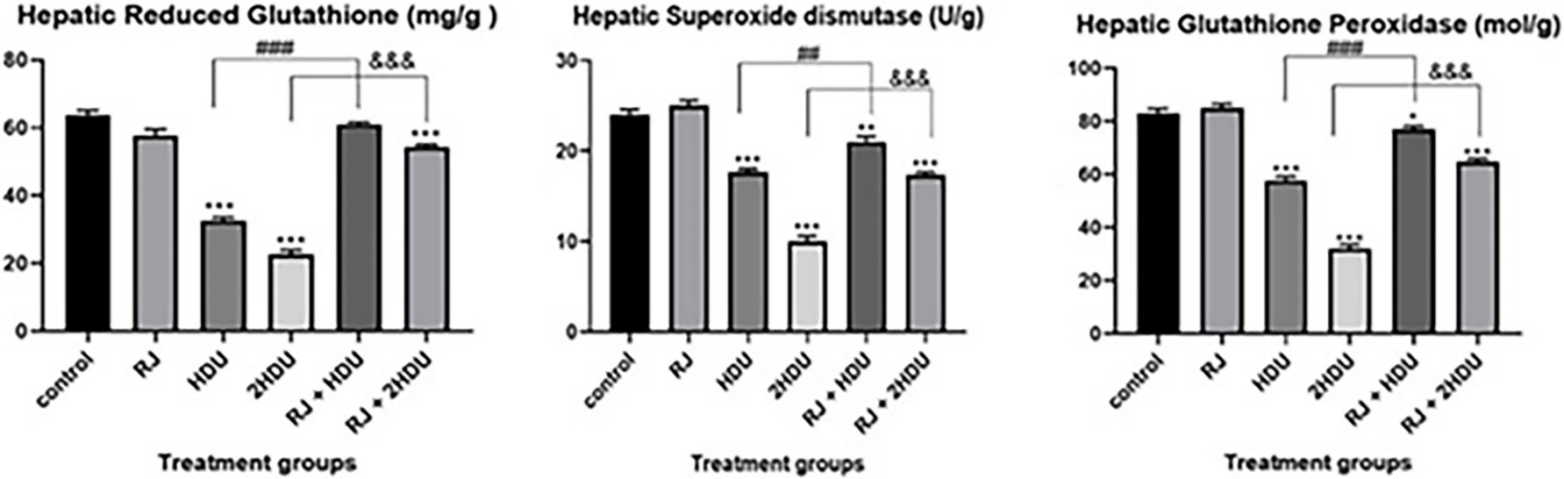
Effects of oral royal jelly (RJ; 100 mg/kg bwt) administration on the anti-oxidant enzymes, reduced glutathione (GSH), glutathione peroxidase (GPx), and superoxide dismutase (SOD) content in rats treated with hydroxyurea (HDU; 225 or 450 mg/kg bwt) for 60 days. Data are presented as the mean ± SEM. Compared to the control, significance at (p < 0.001) *** (ANOVA with Dunnett’s multiple comparison test). Compared to HDU treated groups using Unpaired t-test, significance at p < 0.01 ## and p < 0.001 &&& for HDU and 2HDU groups, respectively).

As shown in Fig 5. HDU caused a significant decline at p < 0.001 in hepatic GSH, SOD, and GPx compared to the control group, especially at the high dose of HDU. Again, RJ administration with a therapeutic dose of HDU significantly alleviated the harmful effect of HDU on the hepatic tissues through elevation of GSH to control levels and increase SOD and GPx content by about 23.5% and 35% but still not reach the control group level (Fig 3). Compared to 2HDU groups, rats receiving RJ with double HDU doses showed significantly (p < 0.001) increased GSH, SOD, and GPx content by 139.3%, 73%, and 100%, respectively, values below the control level.

### 3.5 Apoptosis biomarker (Caspase-3)

**Fig 6:**
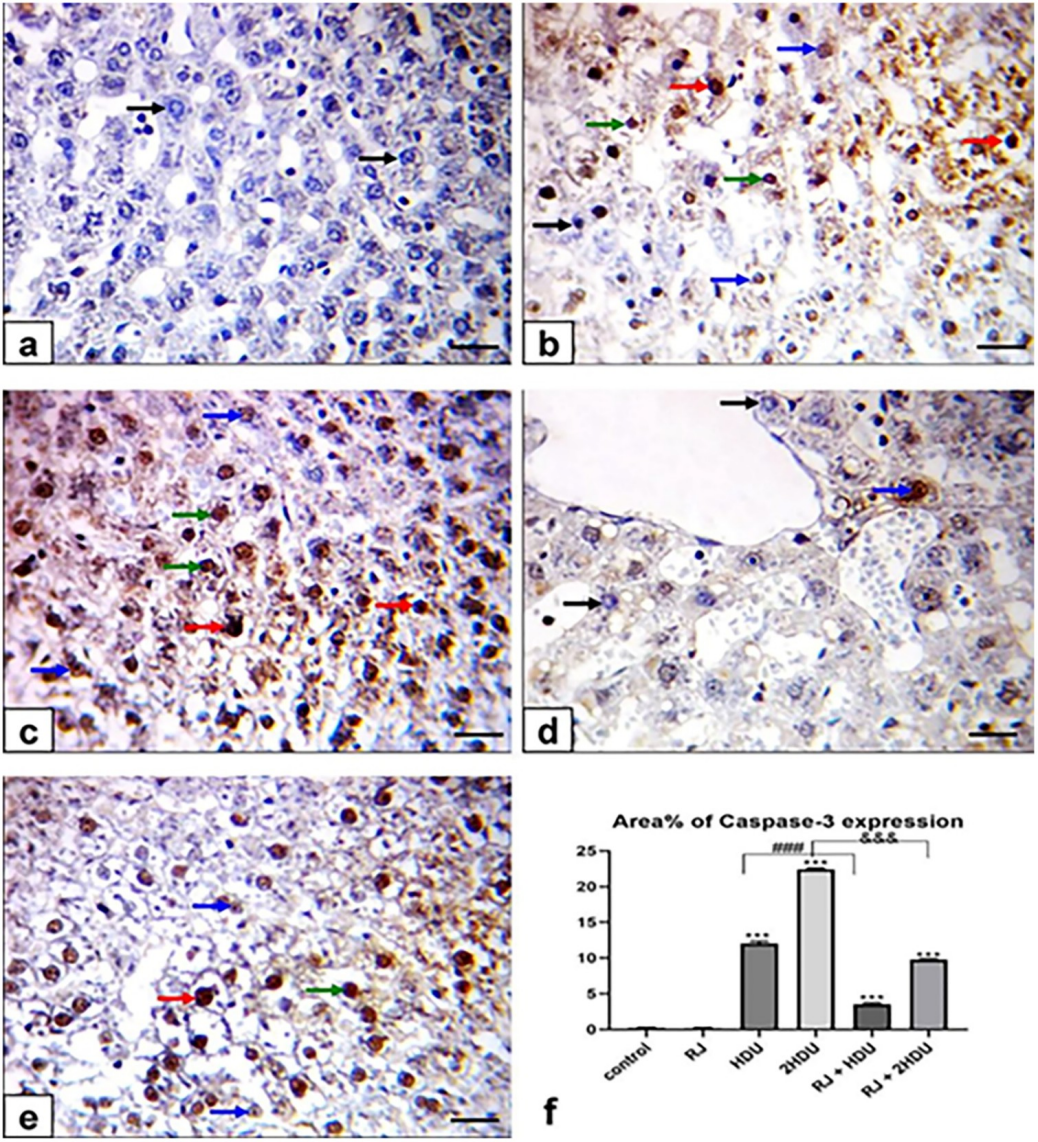
Effects of oral royal jelly (RJ; 100 mg/kg bwt) administration on hepatic caspase-3 protein expression in rats treated with hydroxyurea (HDU; 225 or 450 mg/kg bwt) 60 days. Photomicrograph of Immunohistochemical staining of the hepatic caspase-3 protein expression (scale bar = = 50 μm). (a) control showing negative immunostaining for Caspase-3 protein, (b) HDU (225 mg/kg bwt), (c) 2HDU (450 mg/kg bwt), (d) RJ + HDU and (e) RJ + 2HDU showing nuclear caspase-3 expression in hepatocytes. Negative immunostained hepatocytes (black arrows), strong (red arrows), moderate (green arrows), weak (blue arrows) nuclear caspase-3 expression in hepatocytes. (f) lessening effect RJ on the area (%) of caspase-3 positive cells in the liver of HDU- intoxicated rats. Data are presented as the mean ± SEM. Compared to the control, significance at (p < 0.001) *** (ANOVA with Dunnett’s multiple comparison test). Compared to HDU treated groups using Unpaired t-test, significance at p < 0.001 ### and &&& for HDU and 2HDU groups, respectively.

As illustrated in Fig 6, control and RJ rats revealed negative staining (no expression) for caspase-3 protein expression. The sections from HDU -intoxicated rats exhibited weak to moderate to the positive immune reaction of caspase-3 protein in hepatocytes. Moreover, 2HDU intoxicated rats exhibited moderate to strong brown caspase-3 expression. Oral administration of RJ showing weak caspase-3 expression in HDU and weak to moderate caspase-3 immunostaining in 2HDU intoxicated rats. Approximately 12 ± 0.28% and 22.4 ± 0.16% of hepatic tissues in HDU and 2HDU-treated rats showed strong immunostaining of Caspase-3, respectively. Caspase-3 immunostaining caused by HDU was significantly decreased (p < 0.001) upon administration of RJ.

### 3.6 Pro-inflammatory cytokine (TNF-α)

**Fig 7:**
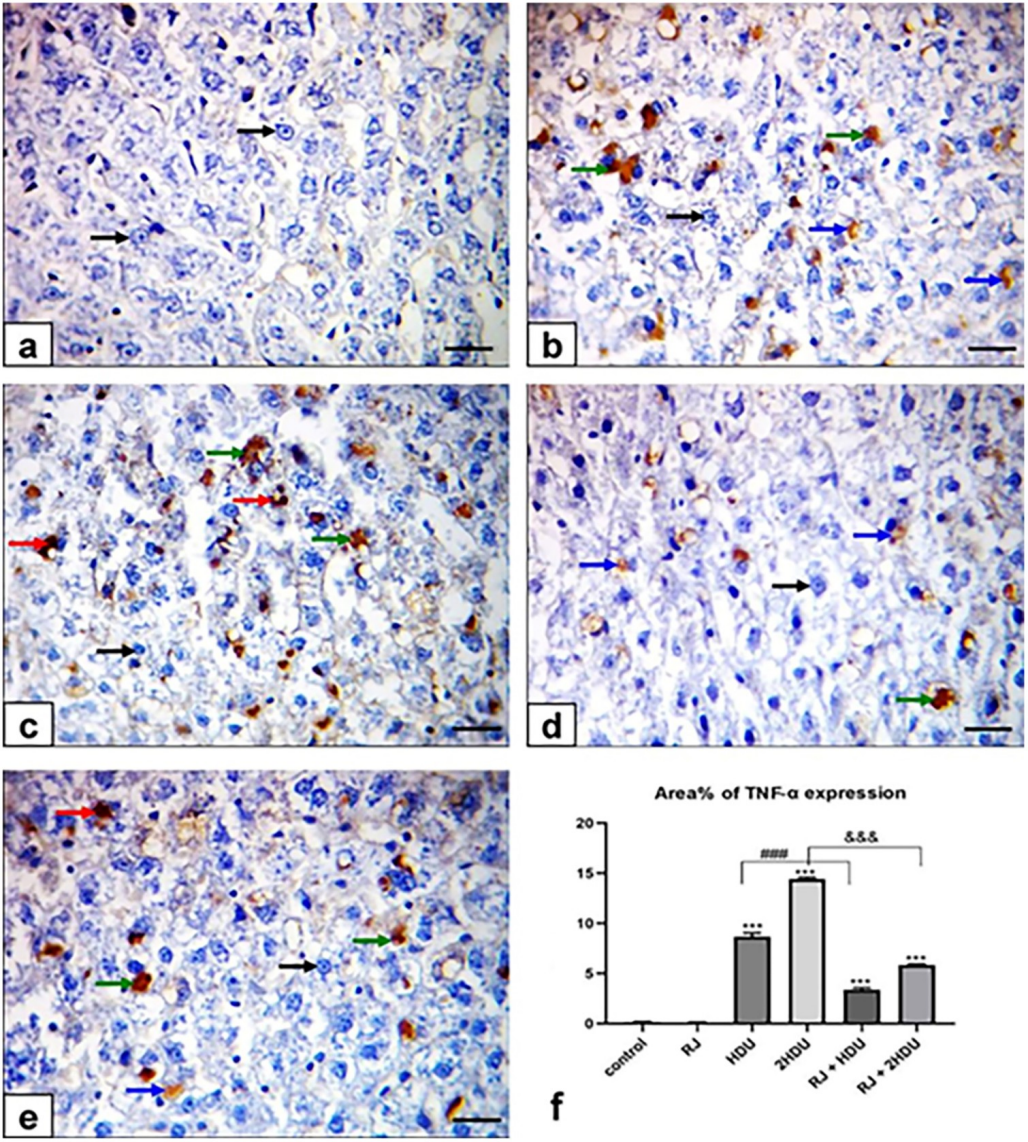
Effects of oral royal jelly (RJ; 100 mg/kg bwt) administration on the pro-inflammatory cytokine (TNF-α) hepatic expression in rats treated with hydroxyurea (HDU; 225 or 450 mg/kg bwt) for 60 days. Photomicrograph of Immunohistochemical staining of the hepatic TNF-α expression (scale bar = = 50 μm). (a) control showing negative immunostaining for TNF-α, (b) HDU (225 mg/kg bwt), (c) 2HDU (450 mg/kg bwt), (d) RJ + HDU and (e) RJ + 2HDU showing nuclear TNF-α expression in hepatocytes and Kupffer cells. Negative immunostained hepatocytes (black arrows), strong (red arrows), moderate (green arrows), weak (blue arrows) nuclear TNF-α expression in hepatocytes and Kupffer cells. (f) Alleviating effect RJ on the area (%) of TNF-α positive hepatocytes and Kupffer cells in HDU- intoxicated rats. Data are presented as the mean ± SEM. Compared to the control, significance at (p < 0.01) ** and (p < 0.001) *** (ANOVA with Dunnett’s multiple comparison test). Compared to HDU treated groups using Unpaired t-test, significance at p < 0.001 ### and &&& for HDU and 2HDU groups, respectively.

The pro-inflammatory cytokine (TNF-α) expressions in different groups are shown in Fig 7. control and RJ rats revealed negative staining for TNF-α expression. The sections from HDU -intoxicated rats exhibited weak to moderate positive immune reactions in hepatocytes. Moreover, 2HDU intoxicated rats exhibited moderate to strong brown expression. Oral administration of RJ showed weak TNF-α expression in HDU and weak to moderate positive immunostaining in 2HDU intoxicated rats. TNF-α Immunostaing area% was demonstrated in about 8.7±0.79% and 14.4±0.38% of hepatic tissues of HDU and 2HDU-treated rats. Again, TNF-α Immunostaining was significantly reduced (p < 0.001) upon RJ administration with the therapeutic and double dose of HDU by about 55.8% and 56.6%, respectively.

### 3.7 Hepatic histopathology

**Fig 8:**
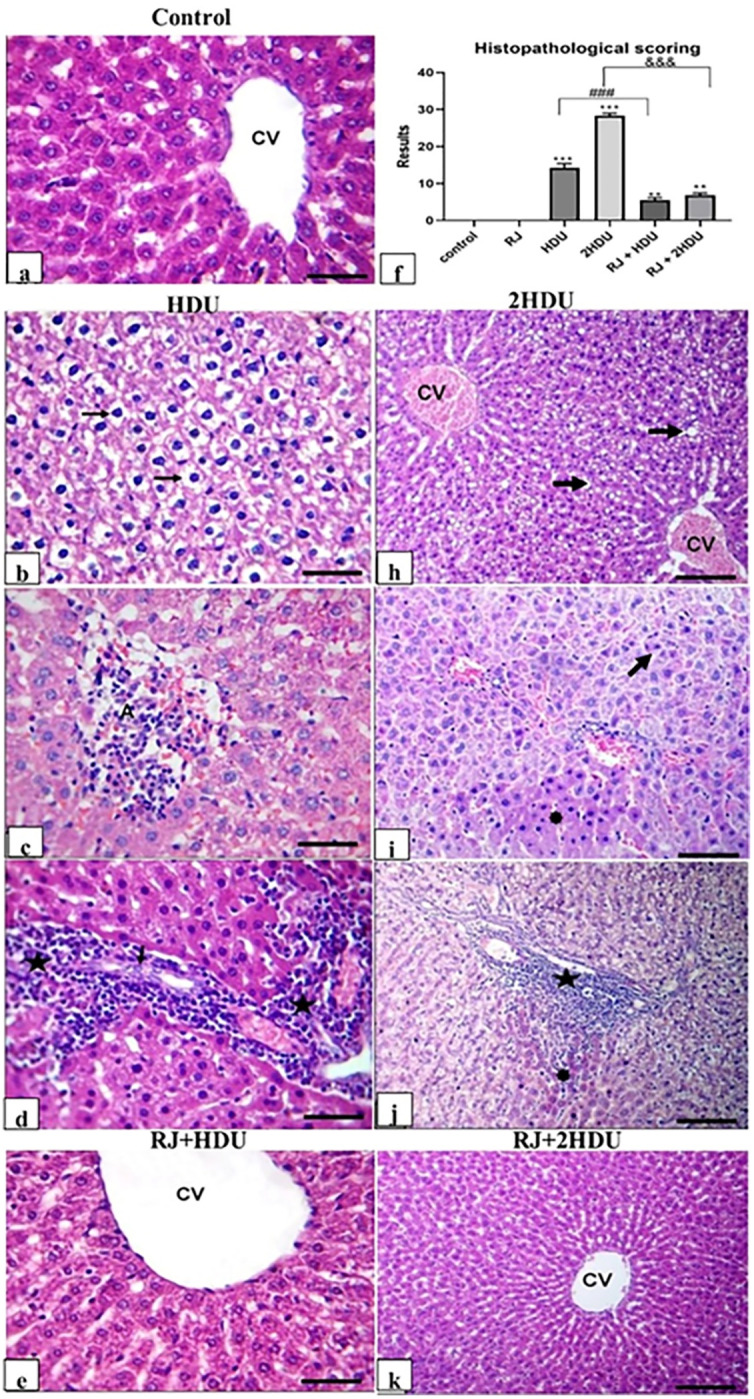
Photomicrograph of rat liver section stained with HE (scale bar = 50 μm): (a) control rats showing normal hepatic histology and architecture. (b, c, d) severe hydropic degeneration of hepatocytes (long black arrow), hepatic necrosis (A), and intense inflammatory cell infiltration in the portal area (black star) with biliary cell degeneration (short black arrow) of rats that received 225 mg /kg bwt HDU daily. h, i, j) severe hydropic degeneration of hepatocytes (long black arrow), apoptotic bodies (short black arrows), congestion of central vein (CV), sharp outline vacuoles (long black arrows), coagulative hepatocellular necrosis (asterisks), intense inflammatory cell infiltration in hepatic necrosis (long black arrows) and in the portal area (black star) in rats that received 450 mg /kg bwt HDU daily In addition, (e) normal hepatic histology and architecture in rats that received a daily dose of 225 mg /kg bwt HDU + 100 mg RJ. (k) multifocal hepatocytes with mild cytoplasmic vacuolation and mild sinusoidal dilatation in rats received a daily dose of 450 mg /kg bwt HDU + 100 mg RJ. (f) Quantitative histopathological scoring.

As shown in Fig 8. Rats treated with a therapeutic dose of HDU exhibited mild to moderate hepatic lesions compared to the control group. The recorded lesions were congestion, moderate to severe hydropic degeneration of hepatocytes, multifocal hepatic necrosis, and inflammatory cell infiltration in a portal with biliary cell degeneration. Meanwhile, rats treated with a double dose of HDU showed moderate to severe hepatic lesions compared to the control group. The hepatic lesions were diffuse mild to severe cellular vacuolation in hydropic degeneration and fatty change. Also, multifocal coagulative hepatocellular necrosis with inflammatory cell infiltration and multifocal hepatocellular apoptotic changes were recorded in all rats treated with 2HDU. Oral royal jelly co-administration shortly after HDU and 2HDU primarily lessen HDU- induced hepatic lesions.

## 4. Discussion

The efficacy of HDU in patients with sickle cell anemia, thalassemia, and various forms of cancer is well documented. However, several side effects have been described as well. Elevation of hepatic enzymes, AST, ALT in patients treated with HDU is one side effect [20, 21]. In the current study, HDU administration, especially at the high dose, exerted hepatotoxic influence in rats via the impairment of hepatic oxidant/antioxidant status, pro-inflammatory cytokine upregulation, apoptosis, and hepatic lesions. In addition, the current study focused on the protective potential of RJ against HDU-induced hepatic injury.

The current data further support previous studies [21, 49] that showed a remarkable elevation of liver function tests (AST, ALT, and ALP) following HDU administration, especially at the high dose of HDU. The HDU- induced hepatic lesions can interpret this obtained result. In our results, the serum liver function tests were improved in RJ co-administrated groups. This obtained result can be interpreted by the ability of RJ to lessen the HDU- induced histopathological changes. The HDU- induced hepatic lesion supported our body weight and hepatosomatic index finding in which we found a significant decrease in body weight and hepatosomatic index in rats treated with HDU in HDU and 2HDU groups compared to the control group, this finding was consistent with [50], they attributed the decreased weight of testes return to the parenchyma atrophy induced by HDU challenge. The body weight and the hepatosomatic index were maintained near the normal level by RJ co-treatment due to the antioxidant activities of RJ [51].

Herein, HDU enhanced ROS production [52] with the incapability of antioxidant defense to completely scavenge them, thus leading to lipid peroxidation, which Corroborates to elevated hepatic MDA level (the end product of lipid peroxidation) [12]. Lipid peroxidation (LPO) causes cellular membrane damage associated with cellular dysfunction and consider as an indicator of oxidative damage [39, 53]. The current data displayed a significant increase of hepatic MDA in HDU treated rats. Additionally, HDU administration significantly increases nitrite levels, Iron-nitrosyl-hemoglobin, and nitrate, proving hydroxyurea’s in vivo metabolism to nitric oxide, a pro-oxidative molecule in the liver [54]. In general, high NO concentration plays a role in apoptosis and oxidative stress, increasing the chance of cell cycle arrest, while low NO concentration stimulates homeostasis and cellular viability [55]. Our results proved that oral administration of RJ with HDU significantly reduced the hepatic concentration of MDA and NO, representing RJ’s antioxidative effect against HDU-induced hepatic injury. This protective effect may be attributed to its antioxidant properties due to the phenolic compounds (flavonoids and cinnamic acid derivatives) [56], the short-chain peptides [57], fatty acids (trans-10-hydroxy-2-decenoic acid), minerals (Fe, Zn, and Cu), some vitamins (A, E, and C) [58] which expressed as a rebalancing of the MDA / GSH, SOD and GPx content in hepatic tissues. This antioxidative ability of RJ can be elucidated by its ability to preventing superoxide anion radical-induced LPO and scavenging free radical-induced oxidative stress.

In the current study, HDU-treated rats showed a significant reduction of hepatic GSH content. This most abundant intracellular non-protein thiol may have been attributed to the ability of HDU to interrupt the redox homeostasis of the liver. Additionally, a static reduction of hepatic SOD activity of HDU treated rats may suggest the interaction of the SOD-active amino acid with free radicals [59]. The susceptibility of the cell membrane to LPO is augmented by the depletion of antioxidants [60]. Oral supplementation of RJ significantly improved hepatic antioxidant status in HDU treated rats by counteracting the toxic effect of HDU on hepatic GSH, SOD, and GPx content. The antioxidative effect of RJ is well-documented in several studies, especially against various toxins-induced testicular injuries [61–63].

HDU toxicity-induced apoptosis, the programmed cell death, is well-documented in the brain [64, 65], testes [66], and lungs [13]. Caspase-3 plays an essential role in the induction of apoptosis [67]. TNF-α is the pro-inflammatory cytokine that increases apoptosis through independent caspase-3 activation [58]. The current data revealed that HDU triggered significant hepatic inflammation and apoptosis via upregulation of TNF-α and caspase-3 apoptotic pathways expressed by the strong immunostaining of both TNF-α and Caspase-3 proteins liver tissues in HDU treated rats. These latter results are in harmony with previous studies [9] and may be explained by the DNA damage due to increased hepatic NO level [68]. In the current study, the downregulation of TNF-α and Caspase-3 expressions in the liver tissues following oral RJ co-administration reflected the RJ’s anti-inflammatory and anti-apoptotic effects against HDU-induced hepatic injury. The RJ’s anti-inflammatory and anti-apoptotic effects were previously reported against impaired fertility due to HDU toxicity in rats [3, 62].

Herein, HDU treated rats exhibited various histopathological changes ranging from mild hepatocellular degeneration to necrosis, attributed to the HDU induced oxidative damage, especially in 2HDU treated rats. Oral RJ administration in HDU and 2HDU treated groups was significantly improved the histopathological changes, which might be mediated via the role of RJ in protecting against inflammatory reactions and scavenging accumulated ROS.

## 5. Conclusion

The current data provided additional information about the hepatotoxicity of hydroxyurea, the widely used antineoplastic medication, especially at the high dose of hydroxyurea. Also, the royal jelly might exert its protective potential against HDU-induced hepatic injury via anti-oxidative, anti-inflammatory, and anti-apoptosis properties. Therefore, the royal jelly can be used as an adjuvant therapy to protect against hydroxyurea-induced hepatic damage.

## Supporting information

S1 File (PDF)

## References

[1] Brose RD, Savonenko A, Devenney B, Smith KD, Reeves RH. Hydroxyurea improves spatial memory and cognitive plasticity in mice and has a mild effect on these parameters in a down syndrome mouse model. Frontiers in aging neuroscience. 2019;11:96. doi: 10.3389/fnagi.2019.00096 31139073 PMC6527804

[2] Banh S, Hales BF. Hydroxyurea exposure triggers tissue-specific activation of p38 mitogen-activated protein kinase signaling and the DNA damage response in organogenesis-stage mouse embryos. toxicological sciences. 2013;133(2):298–308. doi: 10.1093/toxsci/kft069 23492809 PMC3663560

[3] Tohamy HG, El-Karim DRG, El-Sayed YS. Attenuation potentials of royal jelly against hydroxyurea-induced infertility through inhibiting oxidation and release of pro-inflammatory cytokines in male rats. Environmental Science and Pollution Research. 2019;26(21):21524–34. doi: 10.1007/s11356-019-05521-3 31127524

[4] Sun J, Wei Q, Zhou Y, Wang J, Liu Q, Xu H. A systematic analysis of FDA-approved anticancer drugs. BMC Syst Biol. 2017;11(Suppl 5):87-. doi: 10.1186/s12918-017-0464-7 . 28984210 PMC5629554

[5] Chou J, Beck W, Khwaja T, Mayer K, Lien E. Synthesis and Antieancer Activity of Novel Cyclic N-Hydroxyureas. Journal of pharmaceutical sciences. 1977;66(11):1556–61. doi: 10.1002/jps.2600661114 915731

[6] Madaan K, Kaushik D, Verma T. Hydroxyurea: a key player in cancer chemotherapy. Expert review of anticancer therapy. 2012;12(1):19–29. doi: 10.1586/era.11.175 22149429

[7] Lisziewicz J, Foli A, Wainberg M, Lori F. Hydroxyurea in the Treatment of HIV Infection. Drug Saf. 2003;26(9):605–24. doi: 10.2165/00002018-200326090-00002 12814330

[8] Roumenina LT, Chadebech P, Bodivit G, Vieira-Martins P, Grunenwald A, Boudhabhay I, et al. Complement activation in sickle cell disease: dependence on cell density, hemolysis and modulation by hydroxyurea therapy. American journal of hematology. 2020;95(5):456–64. doi: 10.1002/ajh.25742 31990387

[9] Gao W, Jin Y, Hao J, Huang S, Wang D, Quan F, et al. Hydroxyurea affects in vitro porcine oocyte maturation through increased apoptosis and oxidative stress. Biosci Rep. 2021;41(4):BSR20203091. doi: 10.1042/BSR20203091 33844009 PMC8062957

[10] Vickers NJ. Animal communication: when i’m calling you, will you answer too? Curr Biol. 2017;27(14):R713–R5. doi: 10.1016/j.cub.2017.05.064 28743020

[11] Abdul-Baqi MS, Sadiq WS, Omair HAA-H. Evaluation of the Genotoxic Effect of Hydroxyurea Using Cytogenetics Endpointsin White Mice. Annals of the Romanian Society for Cell Biology. 2021;25(6):6092–101.

[12] Ahmad MF, Ansari MO, Jameel S, Parveen N, Siddique HR, Shadab G. Protective role of nimbolide against chemotherapeutic drug hydroxyurea induced genetic and oxidative damage in an animal model. Environ Toxicol Pharmacol. 2018;60:91–9. doi: 10.1016/j.etap.2018.04.006 29679812

[13] Woo G-H, Bak E-J, Nakayama H, Doi K. Hydroxyurea (HU)-induced apoptosis in the mouse fetal lung. Exp Mol Pathol. 2005;79(1):59–67. doi: 10.1016/j.yexmp.2005.02.007 16005713

[14] Woo G, Katayama K, Jung J, Uetsuka K, Bak E, Nakayama H. Hydroxyurea (HU)-induced apoptosis in the mouse fetal tissues. Histol Histopathol. 2003. doi: 10.14670/HH-18.387 12647788

[15] AKÇA H, ÖZEŞ ON. Hydroxyurea induces p53 accumulation and apoptosis in human cervical carcinoma cells. Turkish Journal of Biology. 2002;26(3):145–50.

[16] Teng S, Ma C, Yu Y, Yi C. Hydroxyurea promotes TET1 expression and induces apoptosis in osteosarcoma cells. Biosci Rep. 2019;39(5). doi: 10.1042/BSR20190456 30988069 PMC6522705

[17] Hanft VN, Fruchtman SR, Pickens CV, Rosse WF, Howard TA, Ware RE. Acquired DNA mutations associated with in vivo hydroxyurea exposure. Blood, The Journal of the American Society of Hematology. 2000;95(11):3589–93. 10828048

[18] Oliveira EAMd, Boy KdA, Santos APP, Machado CdS, Velloso-Rodrigues C, Gerheim PSAS, et al. Evaluation of hydroxyurea genotoxicity in patients with sickle cell disease. Einstein (São Paulo). 2019;17(4). 10.31744/einstein_journal/2019AO4742 PMC6750882 31508660

[19] Heddle R, Calvert A. Hydroxyurea induced hepatitis. Med J Aust. 1980;1(3):121-. doi: 10.5694/j.1326-5377.1980.tb134684.x 7374520

[20] Shimizu T, Mori T, Karigane D, Kikuchi T, Koda Y, Toyama T, et al. Hydroxyurea (hydroxycarbamide)-induced hepatic dysfunction confirmed by drug-induced lymphocyte stimulation test. [Rinsho ketsueki] The Japanese journal of clinical hematology. 2014;55(1):125–9. 24492045

[21] Hallam MJ, Kolesar JM. Hydroxyurea induced acute elevations in liver function tests. J Oncol Pharm Pract. 2008;14(1):61–3. doi: 10.1177/1078155207086814 18337443

[22] Snodgrass RE. Anatomy of the honey bee: Cornell University Press; 2018.

[23] Ramanathan ANKG, Nair AJ, Sugunan VS. A review on Royal Jelly proteins and peptides. J Funct Foods. 2018;44:255–64.

[24] Kocot J, Kiełczykowska M, Luchowska-Kocot D, Kurzepa J, Musik I. Antioxidant potential of propolis, bee pollen, and royal jelly: possible medical application. Oxid Med Cell Longev. 2018;2018. 10.1155/2018/7074209 PMC5954854 29854089

[25] Saeed M, Kalhoro S, Naveed M, Hassan F, Umar M, Rashid M, et al. Prospects of royal jelly as a potential natural feed additive in poultry diets. Worlds Poult Sci J. 2018;74(3):499–508.

[26] El-Nekeety AA, El-Kholy W, Abbas NF, Ebaid A, Amra HA, Abdel-Wahhab MA. Efficacy of royal jelly against the oxidative stress of fumonisin in rats. Toxicon. 2007;50(2):256–69. doi: 10.1016/j.toxicon.2007.03.017 17490698

[27] Kanbur M, Eraslan G, Beyaz L, Silici S, Liman BC, Altınordulu Ş, et al. The effects of royal jelly on liver damage induced by paracetamol in mice. Exp Toxicol Pathol. 2009;61(2):123–32. doi: 10.1016/j.etp.2008.06.003 18693095

[28] Uzbekova D, Chugunova L, Makarova V, Ryabkov A, Mirgorodskaya L. Efficacy of royal jelly and lactulose on thyroxin-induced liver damage in rats. J Hepatol. 1998;28:157.9537853

[29] Zimmermann A. Liver regeneration: the emergence of new pathways. Med Sci Monit. 2002;8(3):RA53–RA63. 11887042

[30] Care IoLARCo, Animals UoL. Guide for the care and use of laboratory animals: US Department of Health and Human Services, Public Health Service, National …; 1986.

[31] Amirshahi T, Najafi G, Nejati V. Protective effect of royal jelly on fertility and biochemical parameters in bleomycin- induced male rats. Iranian journal of reproductive medicine. 2014;12(3):209. 24799882 PMC4009575

[32] Manas GE, Najafi G. Protective effects of royal jelly on the histomorphologic, oxidative stress and sperm parameters in Ofloxacin treated rat. Comparative clinical pathology. 2017;26(5):1111–5.

[33] Nair AB, Jacob S. A simple practice guide for dose conversion between animals and human. Journal of basic and clinical pharmacy. 2016;7(2):27. doi: 10.4103/0976-0105.177703 27057123 PMC4804402

[34] Liu IM, Hsu FL, Chen CF, Cheng JT. Antihyperglycemic action of isoferulic acid in streptozotocin-induced diabetic rats. British Journal of Pharmacology. 2000;129(4):631–6. doi: 10.1038/sj.bjp.0703082 10683186 PMC1571880

[35] Liu I-M, Tzeng T-F, Liou S-S, Lan T-W. Myricetin, a naturally occurring flavonol, ameliorates insulin resistance induced by a high-fructose diet in rats. Life sciences. 2007;81(21–22):1479–88. doi: 10.1016/j.lfs.2007.08.045 17976658

[36] Vermeij J-D, Aslami H, Fluiter K, Roelofs JJ, van den Bergh WM, Juffermans NP, et al. Traumatic brain injury in rats induces lung injury and systemic immune suppression. Journal of neurotrauma. 2013;30(24):2073–9. doi: 10.1089/neu.2013.3060 23937270

[37] Reitman S, Frankel S. A colorimetric method for the determination of serum glutamic oxalacetic and glutamic pyruvic transaminases. Am J Clin Pathol. 1957;28(1):56–63. doi: 10.1093/ajcp/28.1.56 13458125

[38] Belfield A, Goldberg D. Revised assay for serum phenyl phosphatase activity using 4-amino-antipyrine. Enzyme. 1971;12:561–73. doi: 10.1159/000459586 5169852

[39] Hassan E, El-Neweshy M, Hassan M, Noreldin A. Thymoquinone attenuates testicular and spermotoxicity following subchronic lead exposure in male rats: Possible mechanisms are involved. Life sciences. 2019;230:132–40. doi: 10.1016/j.lfs.2019.05.067 31136753

[40] Ohkawa H, Ohishi N, Yagi K. Assay for lipid peroxides in animal tissues by thiobarbituric acid reaction. Anal Biochem. 1979;95(2):351–8. doi: 10.1016/0003-2697(79)90738-3 36810

[41] Koltuksuz U, Irmak MK, Karaman A, Uz E, Var A, Özyurt H, et al. Testicular nitric oxide levels after unilateral testicular torsion/detorsion in rats pretreated with caffeic acid phenethyl ester. Urol Res. 2000;28(6):360–3. doi: 10.1007/s002400000145 11221913

[42] Beutler E. Improved method for the determination of blood glutathione. J Lab Clin Med. 1963;61:882–8. 13967893

[43] Paglia DE, Valentine WN. Studies on the quantitative and qualitative characterization of erythrocyte glutathione peroxidase. The Journal of laboratory and clinical medicine. 1967;70(1):158–69. 6066618

[44] Nishikimi M, Rao NA, Yagi K. The occurrence of superoxide anion in the reaction of reduced phenazine methosulfate and molecular oxygen. Biochem Biophys Res Commun. 1972;46(2):849–54. doi: 10.1016/s0006-291x(72)80218-3 4400444

[45] Hassan E, Kahilo K, Kamal T, El-Neweshy M, Hassan M. Protective effect of diallyl sulfide against lead-mediated oxidative damage, apoptosis and down-regulation of CYP19 gene expression in rat testes. Life Sci. 2019;226:193–201. doi: 10.1016/j.lfs.2019.04.020 30986445

[46] Varghese F, Bukhari AB, Malhotra R, De A. IHC Profiler: an open source plugin for the quantitative evaluation and automated scoring of immunohistochemistry images of human tissue samples. PLoS One. 2014;9(5):e96801. doi: 10.1371/journal.pone.0096801 24802416 PMC4011881

[47] Young K, Morrison H. Quantifying microglia morphology from photomicrographs of immunohistochemistry prepared tissue using ImageJ. Journal of visualized experiments: JoVE. 2018;(136). doi: 10.3791/57648 29939190 PMC6103256

[48] Culling CFA. Handbook of histopathological and histochemical techniques: including museum techniques: Butterworth-Heinemann; 2013.

[49] Opoka RO, Ndugwa CM, Latham TS, Lane A, Hume HA, Kasirye P, et al. Novel use Of Hydroxyurea in an African Region with Malaria (NOHARM): a trial for children with sickle cell anemia. Blood, The Journal of the American Society of Hematology. 2017;130(24):2585–93. 10.1182/blood-2017-06-788935 29051184

[50] Saalu L, Jewo P, Yama O, Oguntola J. Evaluation of the histomorphometric evidences of hydroxyurea-induced testicular cytotoxicity in sprague-dawley rat. J Pharmacol Toxicol. 2011;6:409–17.

[51] Almeer RS, AlBasher GI, Alarifi S, Alkahtani S, Ali D, Moneim AEA. Royal jelly attenuates cadmium-induced nephrotoxicity in male mice. Sci Rep. 2019;9(1):1–12. 30967588 10.1038/s41598-019-42368-7 PMC6456607

[52] Cokic VP, Smith RD, Beleslin-Cokic BB, Njoroge JM, Miller JL, Gladwin MT, et al. Hydroxyurea induces fetal hemoglobin by the nitric oxide–dependent activation of soluble guanylyl cyclase. The Journal of clinical investigation. 2003;111(2):231–9. doi: 10.1172/JCI16672 12531879 PMC151872

[53] Khafaga AF, El-Sayed YS. All-trans-retinoic acid ameliorates doxorubicin-induced cardiotoxicity: in vivo potential involvement of oxidative stress, inflammation, and apoptosis via caspase-3 and p53 down-expression. Naunyn-Schmiedeberg’s archives of pharmacology. 2018;391(1):59–70. doi: 10.1007/s00210-017-1437-5 29085977

[54] Huang J, Yakubu M, Kim-Shapiro DB, King SB. Rat liver-mediated metabolism of hydroxyurea to nitric oxide. Free Radic Biol Med. 2006;40(9):1675–81. doi: 10.1016/j.freeradbiomed.2006.01.002 16632127

[55] Thomas DD, Ridnour LA, Isenberg JS, Flores-Santana W, Switzer CH, Donzelli S, et al. The chemical biology of nitric oxide: implications in cellular signaling. Free Radic Biol Med. 2008;45(1):18–31. doi: 10.1016/j.freeradbiomed.2008.03.020 18439435 PMC2572721

[56] Kolayli S, Sahin H, Can Z, Yildiz O, Malkoc M, Asadov A. A member of complementary medicinal food: anatolian royal jellies, their chemical compositions, and antioxidant properties. J Evid Based Complementary Altern Med. 2016;21(4):NP43–NP8. doi: 10.1177/2156587215618832 26620573

[57] Guo H, Ekusa A, Iwai K, Yonekura M, Takahata Y, Morimatsu F. Royal jelly peptides inhibit lipid peroxidation in vitro and in vivo. J Nutr Sci Vitaminol (Tokyo). 2008;54(3):191–5. doi: 10.3177/jnsv.54.191 18635904

[58] Alvarez-Suarez JM. Bee products-chemical and biological properties: Springer; 2017.

[59] Xin Z, Zhang X, Hu W, Tan DX, Han M, Ji T, et al. The protective effects of melatonin on organisms against the environmental pollutants of heavy metal and non-metal toxins. Melatonin Research. 2019;2(4):99–120.

[60] Tohamy HG, El Okle OS, Goma AA, Abdel-Daim MM, Shukry M. Hepatorenal protective effect of nano-curcumin against nano-copper oxide-mediated toxicity in rats: Behavioral performance, antioxidant, anti-inflammatory, apoptosis, and histopathology. Life Sciences. 2022:120296. doi: 10.1016/j.lfs.2021.120296 35045342

[61] Asadi N, Kheradmand A, Gholami M, Saidi SH, Mirhadi SA. Effect of royal jelly on testicular antioxidant enzymes activity, MDA level and spermatogenesis in rat experimental Varicocele model. Tissue Cell. 2019;57:70–7. doi: 10.1016/j.tice.2019.02.005 30947966

[62] Azad F, Nejati V, Shalizar-Jalali A, Najafi G, Rahmani F. Antioxidant and anti-apoptotic effects of royal jelly against nicotine-induced testicular injury in mice. Environ Toxicol. 2019;34(6):708–18. doi: 10.1002/tox.22737 30896085

[63] Park MJ, Kim BY, Deng Y, Park HG, Choi YS, Lee KS, et al. Antioxidant capacity of major royal jelly proteins of honeybee (Apis mellifera) royal jelly. Journal of Asia-Pacific Entomology. 2020;23(2):445–8.

[64] Molina V, Rodríguez-Vázquez L, Martí J. Patterns of apoptosis and autophagy activation after hydroxyurea exposure in the rat cerebellar external granular layer: An immunoperoxidase and ultrastructural analysis. Neurotox Res. 2020;37(1):93–9. doi: 10.1007/s12640-019-00094-y 31410685

[65] Rodríguez-Vázquez L, Vons O, Valero O, Martí J. Hydroxyurea exposure and development of the cerebellar external granular layer: Effects on granule cell precursors, bergmann glial and microglial cells. Neurotox Res. 2019;35(2):387–400. doi: 10.1007/s12640-018-9964-5 30276718

[66] Zhou L, Wu C-q, Luo Y-w, Liao M-y, Sun Z-y. Studies on the characteristics and mechanisms of testicular toxicity induced by Hydroxyurea. Toxicol Mech Methods. 2015;25(5):396–401. doi: 10.3109/15376516.2015.1045657 26399158

[67] Degterev A, Boyce M, Yuan J. A decade of caspases. Oncogene. 2003;22(53):8543–67. doi: 10.1038/sj.onc.1207107 14634618

[68] Wang C, Gong G, Sheh A, Muthupalani S, Bryant E, Puglisi D, et al. Interleukin-22 drives nitric oxide-dependent DNA damage and dysplasia in a murine model of colitis-associated cancer. Mucosal Immunol. 2017;10(6):1504–17. doi: 10.1038/mi.2017.9 28198364 PMC5557711

