# Supplementary figures and images for: Protective potential of royal jelly against hydroxyurea -induced hepatic injury in rats via antioxidant, anti-inflammatory, and anti-apoptosis properties
Source: PLoS One. 2022 Mar 18;17(3):e0265261. doi: 10.1371/journal.pone.0265261 (PMC8932593; doi:10.1371/journal.pone.0265261)

|     | control | RJ | HDU | 2HDU | RJ + HDU | RJ + 2HDU |
|-----|---------|----|-----|------|----------|-----------|
| AST | 54      | 53 | 99  | 204  | 68       | 120       |
|     | 65      | 55 | 120 | 255  | 99       | 118       |
|     | 45      | 58 | 127 | 207  | 68       | 137       |
|     | 55      | 67 | 105 | 198  | 85       | 101       |
|     | 52      | 62 | 165 | 189  | 55       | 145       |
| ALT | 56      | 77 | 129 | 220  | 88       | 124       |
|     | 54      | 65 | 125 | 245  | 84       | 165       |
|     | 67      | 67 | 142 | 207  | 73       | 123       |
|     | 81      | 48 | 175 | 298  | 105      | 112       |
|     | 57      | 60 | 156 | 301  | 99       | 99        |
| ALP | 65      | 80 | 138 | 305  | 90       | 157       |
|     | 77      | 78 | 187 | 355  | 102      | 143       |
|     | 80      | 67 | 165 | 288  | 85       | 134       |
|     | 82      | 68 | 178 | 249  | 79       | 128       |
|     | 67      | 57 | 150 | 265  | 98       | 168       |
| MDA | 45      | 45 | 66  | 96   | 52       | 61        |
|     | 46      | 45 | 66  | 95   | 50       | 60        |
|     | 44      | 43 | 72  | 99   | 54       | 59        |
|     | 48      | 44 | 77  | 103  | 53       | 62        |
|     | 49      | 44 | 69  | 97.5 | 52.5     | 60.5      |
| GSH | 61      | 59 | 33  | 22   | 60       | 55        |
|     | 64      | 54 | 31  | 25   | 61       | 53        |
|     | 66      | 60 | 34  | 21   | 62       | 55        |
|     | 64      | 59 | 33  | 22   | 61       | 55        |
|     | 64      | 58 | 32  | 21   | 60       | 54        |
| NO  | 67      | 69 | 90  | 110  | 75       | 86        |
|     | 68      | 70 | 89  | 115  | 77       | 82        |
|     | 70      | 72 | 85  | 118  | 79       | 85        |
|     | 69      | 73 | 84  | 116  | 77       | 84        |
|     | 70      | 70 | 87  | 117  | 78       | 83        |
| SOD | 23      | 24 | 18  | 10   | 21       | 18        |
|     | 25      | 26 | 17  | 11   | 20       | 17        |
|     | 24      | 25 | 18  | 9    | 22       | 17        |
|     | 22      | 26 | 19  | 8    | 20       | 19        |
|     | 25      | 25 | 16  | 6    | 22       | 17        |
| GPX | 80      | 82 | 55  | 35   | 75       | 64        |

|  |    |    |    |    |    |    |
|--|----|----|----|----|----|----|
|  | 83 | 86 | 58 | 30 | 77 | 63 |
|  | 86 | 87 | 60 | 31 | 79 | 67 |
|  | 85 | 83 | 60 | 29 | 80 | 63 |
|  | 84 | 85 | 57 | 30 | 74 | 65 |

|          |   |   |      |      |     |     |
|----------|---|---|------|------|-----|-----|
| NECROSIS | 0 | 0 | 15.3 | 27   | 5.1 | 5.1 |
|          | 0 | 0 | 13.2 | 29.2 | 6.2 | 6.8 |
|          | 0 | 0 | 14.1 | 30.2 | 3.5 | 8.5 |
|          | 0 | 0 | 11.1 | 28.4 | 7.4 | 6.5 |
|          | 0 | 0 | 17.6 | 26.9 | 5.5 | 7.5 |

|               |     |     |     |      |     |     |
|---------------|-----|-----|-----|------|-----|-----|
| TNF- $\alpha$ | 0.1 | 0.1 | 9.7 | 14.2 | 3.3 | 6.1 |
|               | 0.4 | 0.3 | 9.1 | 14.6 | 3.1 | 5.7 |
|               | 0.2 | 0.1 | 8.8 | 13.9 | 4   | 5.5 |
|               | 0   | 0   | 8.1 | 14.9 | 3.6 | 6   |
|               | 0   | 0   | 7.7 | 14.5 | 2.9 | 5.9 |

|           |     |     |     |     |     |     |
|-----------|-----|-----|-----|-----|-----|-----|
| Body weig | 485 | 482 | 410 | 395 | 440 | 433 |
|           | 482 | 479 | 415 | 385 | 442 | 434 |
|           | 483 | 480 | 422 | 400 | 443 | 435 |
|           | 480 | 481 | 423 | 380 | 441 | 432 |
|           | 482 | 479 | 416 | 393 | 442 | 431 |

|     |          |          |          |          |          |          |
|-----|----------|----------|----------|----------|----------|----------|
| HIS | 2.903093 | 2.834025 | 2.688889 | 2.324706 | 2.920455 | 2.849885 |
|     | 3.112033 | 2.82881  | 2.721239 | 2.247059 | 2.923077 | 2.894009 |
|     | 3.00207  | 2.914583 | 2.453    | 2.178571 | 2.808126 | 2.415    |
|     | 3.00207  | 2.7125   | 2.64155  | 2.1458   | 2.75421  | 2.1456   |
|     | 3.0014   | 2.831418 | 2.66522  | 2.212815 | 2.864291 | 2.632443 |

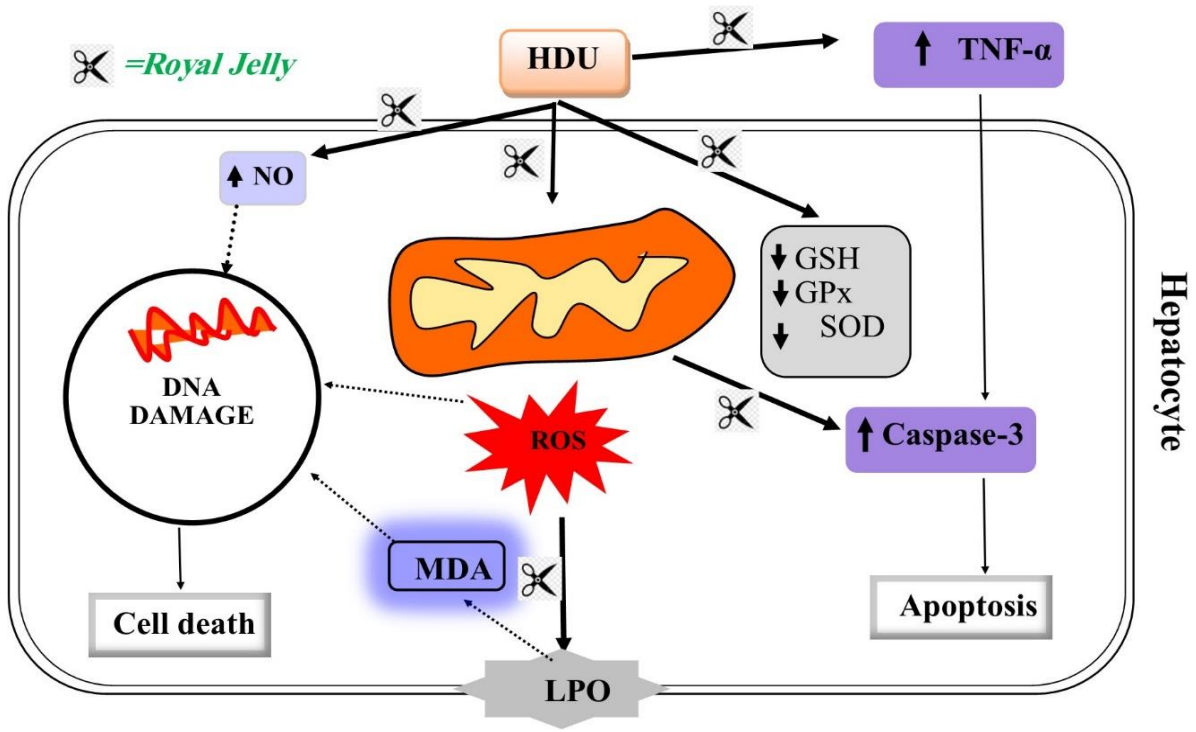

Supplement: S1 File — (PDF) [file pone.0265261.s001.pdf]
